# ^68^Ga-DOTA-exendin-4 PET/CT for insulinoma localization in patients with negative or inconclusive conventional imaging

**DOI:** 10.1210/clinem/dgag214

**Published:** 2026-05-21

**Authors:** Julia G Fricke, Katharina Laubner, Manuela Schildmeijer, Lea Menz, Camilla Wojsa, Philipp T Meyer, Jochen Seufert, Damian Wild, Emanuel Christ, Martin T Freitag

**Affiliations:** Division of Nuclear Medicine, University Hospital Basel, 4031 Basel, Switzerland; Division of Endocrinology and Diabetology, Department of Medicine II, Medical Center—University of Freiburg, Faculty of Medicine, University of Freiburg, 79106 Freiburg, Germany; ENETS Center of Excellence for Neuroendocrine and Endocrine Tumors, University Medical Center Freiburg, Faculty of Medicine, University of Freiburg, 79106 Freiburg, Germany; Berta-Ottenstein Program, Faculty of Medicine University of Freiburg, 79106 Freiburg, Germany; Division of Nuclear Medicine, University Hospital Basel, 4031 Basel, Switzerland; Division of Endocrinology and Diabetology, Department of Medicine II, Medical Center—University of Freiburg, Faculty of Medicine, University of Freiburg, 79106 Freiburg, Germany; Division of Nuclear Medicine, University Hospital Basel, 4031 Basel, Switzerland; ENETS Center of Excellence for Neuroendocrine and Endocrine Tumors, University Medical Center Freiburg, Faculty of Medicine, University of Freiburg, 79106 Freiburg, Germany; Department of Nuclear Medicine, Medical Center—University of Freiburg, Faculty of Medicine, University of Freiburg, 79106 Freiburg, Germany; Division of Endocrinology and Diabetology, Department of Medicine II, Medical Center—University of Freiburg, Faculty of Medicine, University of Freiburg, 79106 Freiburg, Germany; ENETS Center of Excellence for Neuroendocrine and Endocrine Tumors, University Medical Center Freiburg, Faculty of Medicine, University of Freiburg, 79106 Freiburg, Germany; Division of Nuclear Medicine, University Hospital Basel, 4031 Basel, Switzerland; ENETS Center of Excellence for Neuroendocrine and Endocrine Tumors, University Hospital Basel, 4031 Basel, Switzerland; ENETS Center of Excellence for Neuroendocrine and Endocrine Tumors, University Hospital Basel, 4031 Basel, Switzerland; Division of Endocrinology, University Hospital Basel, 4031 Basel, Switzerland; ENETS Center of Excellence for Neuroendocrine and Endocrine Tumors, University Medical Center Freiburg, Faculty of Medicine, University of Freiburg, 79106 Freiburg, Germany; Department of Nuclear Medicine, Medical Center—University of Freiburg, Faculty of Medicine, University of Freiburg, 79106 Freiburg, Germany

**Keywords:** GLP-1 receptor, insulinoma, GLP-1 PET/CT, [^68^Ga]Ga-DOTA-exendin-4 PET/CT, molecular imaging, hyperinsulinism

## Abstract

**Context:**

Insulinomas are the main cause of endogenous hyperinsulinemic hypoglycemia (EHH) in adults, with surgery as the only cure. Pancreas-preserving surgery is preferred, making precise preoperative localization crucial. GLP-1R-targeted imaging, such as [^68^Ga]Ga-DOTA-exendin-4 PET/CT, outperforms other imaging procedures for insulinoma localization.

**Objective:**

To investigate the performance and clinical impact of [^68^Ga]Ga-DOTA-exendin-4 PET/CT for the detection of insulinomas in patients with EHH and prior negative or inconclusive conventional imaging.

**Methods:**

In this retrospective, real-world, dual-center imaging study, 101 patients with biochemically proven EHH and/or a positive Whipple's triad underwent [^68^Ga]Ga-DOTA-exendin-4 PET/CT at two tertiary centers between April 2017 and March 2024. Among these, 58 patients with prior negative or inconclusive conventional imaging constituted the primary analysis cohort. Endpoints: Insulinoma detection rate and sensitivity by [^68^Ga]Ga-DOTA-exendin-4 PET/CT after negative or inconclusive conventional imaging and impact on clinical management.

**Results:**

Among the 58 patients with prior negative or inconclusive conventional imaging, [^68^Ga]Ga-DOTA-exendin-4 PET/CT was positive in 42 patients, corresponding to a detection rate of 72% (95% CI, 59-83%). Thirty patients subsequently underwent PET/CT-guided surgery. Overall, 32 patients had histological verification (including two PET/CT-negative cases), corresponding to a sensitivity of 93.8% (95% CI, 79-99%).

**Conclusion:**

In real-world clinical practice, [^68^Ga]Ga-DOTA-exendin-4 PET/CT provides a high detection rate of 72% for localizing insulinomas, including one case of diffuse nesidioblastosis in patients with EHH and prior negative or inconclusive conventional imaging, thereby enabling PET/CT-guided surgical approaches. These findings underscore the substantial clinical impact of GLP-1R-targeted PET/CT on patient management and prognosis.

Insulinomas represent the most prevalent cause of endogenous hyperinsulinemic hypoglycemia (EHH). They are rare neuroendocrine tumors, usually benign (1), most often presenting as solitary lesions; when occurring as multiple tumors, they are more frequently associated with hereditary syndromes such as multiple endocrine neoplasia type 1 (2). Rare entities of EHH are represented by different types of congenital hyperinsulinism, a group of genetic disorders that usually become evident in childhood, but milder clinical manifestations may first be discovered in adults (3) or can be acquired in adulthood. In this context, diffuse or focal β-cell hyperplasia and hypertrophy within the islets of Langerhans can be observed, a pattern historically referred to as nesidioblastosis (4, 5).

Patients with EHH typically present symptoms that meet the criteria of Whipple's triad, which includes (i) signs and symptoms of hypoglycemia, (ii) documented low plasma glucose levels during episodes, and (iii) relief of symptoms after glucose administration. Biochemical diagnosis of EHH is based on various endocrine tests, especially the 72-h fasting test (6, 7). Surgery remains the usual curative treatment for insulinoma, with preference for pancreas-preserving approaches such as limited segmental resection or enucleation to minimize postoperative complications (8, 9). Alternatively, newer data indicate that local-ablative interventions based on endosonography with ethanol ablation or radio-frequency ablation can be a successful individual therapeutic approach, particularly in patients with relevant comorbidities (10, 11).

Importantly, for planning of any therapeutic intervention accurate preoperative identification and localization of the underlying cause of EHH is critical. Despite advances in radiological cross-sectional imaging, accurate localization of insulinomas remains challenging due to their small size and variable presentation on contrast-enhanced imaging (12), with reported sensitivities of up to 70% for CT, 90% for MRI, and 75% for endoscopic ultrasound (EUS) (13, 14). Consequently, many patients still require additional diagnostic procedures when conventional imaging remains inconclusive. Invasive methods such as angiography with selective arterial calcium stimulation and venous sampling (ASVS) represent a well-established diagnostic option. Several large case series and retrospective studies have demonstrated sensitivities exceeding 85% for insulinoma detection (15-17). However, this approach allows only indirect functional localization within a pancreatic arterial territory, without direct lesion visualization, and is associated with procedure-related risks and potential complications (15, 16).

Somatostatin receptor (SSTR)-targeted PET/CT is commonly used for imaging low-grade neuroendocrine tumors, but its performance in insulinoma is variable, with reported detection rates ranging from 33% to 85% (18, 19). This variability is reflected by low SSTR expression of these tumors on average, hampering detection of small insulinomas (20). Some centers also employ [^18^F]FDOPA PET/CT, though data are limited, and detection rates are even more inconsistent (21, 22).

The glucagon-like peptide-1 receptor (GLP-1R) is usually highly expressed in differentiated insulinomas as well as in β-cell hyperplasia and hypertrophy within the islets of Langerhans (histologically referred to as nesidioblastosis) and in normal pancreatic β-cells (23). In a prospective intraindividual comparison, GLP-1R-targeted PET/CT using the highly specific ligand exendin-4 ([^68^Ga]Ga-DOTA-exendin-4, [^68^Ga]Ga-NOTA-exendin-4, and [^68^Ga]Ga-NODAGA-exendin-4) PET/CT demonstrated markedly higher sensitivity for the detection of benign insulinomas compared with conventional cross-sectional imaging. While GLP-1R-targeted PET/CT achieved sensitivities of 95% and 98%, respectively, contrast-enhanced MRI and CT reached only 69% and 74% (18, 24, 25). Accordingly, the European Neuroendocrine Tumor Society (ENETS) guidelines recommend GLP-1R-targeted imaging or ASVS as complementary localization techniques in selected patients with EHH when conventional imaging or EUS is negative or inconclusive (26).

While the above-mentioned prospective studies support the diagnostic value of GLP-1R-targeted PET/CT in a controlled setting, its performance in daily clinical practice remains less well characterized. Furthermore, robust evidence is limited for patients with negative or inconclusive conventional imaging, a group of patients that poses the greatest diagnostic challenges. To date, real-world evidence in this specific patient population is scarce. Only Aveline et al have evaluated [^68^Ga]Ga-NODAGA-exendin-4 PET/CT in a real-world cohort with clinically suspected insulinoma and inconclusive/negative prior conventional imaging, reporting a high positive predictive value and a substantial impact on clinical management (27). We, therefore, aimed at assessing real-world data for the detection rate of insulinoma with [^68^Ga]Ga-DOTA-exendin-4 PET/CT in patients with proven EHH including Whipple's triad and negative or inconclusive results on prior conventional imaging in two independent centers in two different countries. The main outcomes include (i) the detection rate of insulinomas in this particularly challenging patient group and (ii) the investigation of the impact of [^68^Ga]Ga-DOTA-exendin-4 PET/CT on subsequent patient management pathways in daily practice.

## Materials and methods

### Study population

In this international, dual-center, retrospective, real-world imaging study, 101 consecutive patients with biochemically proven EHH, confirmed by a fasting test demonstrating inappropriately elevated insulin levels and/or a positive Whipple's triad, who underwent [^68^Ga]Ga-DOTA-exendin-4 PET/CT between April 2017 and March 2024 were included. [^68^Ga]Ga-DOTA-exendin-4 PET/CT was performed at two tertiary centers in two different countries (University Hospital Freiburg, Germany; University Hospital Basel, Switzerland) in the context of routine clinical care. Exclusion criteria were aborted or incomplete [^68^Ga]Ga-DOTA-exendin-4 scans and age < 18 years. This study was approved by the ethics committees of Freiburg (Baden-Württemberg, Germany; ID 24-1492-S1-retro) and Northwest/Central Switzerland (ID 2024_00202).

### Procedures

[^68^Ga]Ga-DOTA-exendin-4 PET/CT was performed either on a GEMINI TF 64 PET/CT or a digital Vereos PET/CT (Philips Healthcare) system at University Hospital Freiburg, Germany and on a PET/16-detector CT scanner (Discovery ST; Siemens Healthineers) at University Hospital Basel, Switzerland. Patients received an intravenous administration of 96.9 ± 26.2 MBq (range, 42-156 MBq), peptide amount 8-31 µg, and [^68^Ga]Ga-DOTA-exendin-4. To minimize the risk of hypoglycemia, a glucose infusion (10-20%, as needed) was administered over 1-2 hours. Subsequently, a single-bed position PET acquisition of the upper abdomen was performed 2 hours postinjection at a rate of 0.3 mm/sec (Basel) or with a scan duration of 5 minutes per bed position (Freiburg). Patients underwent additional contrast-enhanced CT of the upper abdomen to provide an anatomic reference (120 kVp, 30-100 mAs), if no prior CT scan was available.

### Imaging evaluation, histology assessment and follow-up

Negative or inconclusive conventional imaging was defined as the absence of a clearly localizing lesion on contrast-enhanced CT, MRI, and/or endoscopic ultrasonography prior to [^68^Ga]Ga-DOTA-exendin-4 PET/CT. [^68^Ga]Ga-DOTA-exendin-4 PET/CT scans were interpreted in routine clinical practice by one nuclear medicine resident and one board-certified senior nuclear medicine physician, both of whom had access to prior imaging and relevant clinical information.

Subsequent surgical management after [^68^Ga]Ga-DOTA-exendin-4 PET/CT was assessed, including documentation of surgical intervention, procedure type where available, and concordance between [^68^Ga]Ga-DOTA-exendin-4 PET/CT-based lesion localization and intraoperative and histopathological findings.

Patients were personally contacted at least 6 months after [^68^Ga]Ga-DOTA-exendin-4 PET/CT to assess their clinical outcome. As an objective clinical follow-up parameter, a hypoglycemia score was evaluated as published previously (28): 0 indicating no hypoglycemic episodes, 1 indicating manageable episodes treated conservatively such as dietary adjustments, and 2 indicating severe episodes requiring hospitalization or associated with hypoglycemic coma.

### Clinical impact on management

The clinical impact of [^68^Ga]Ga-DOTA-exendin-4 PET/CT was assessed descriptively. Specifically, it was recorded whether positive [^68^Ga]Ga-DOTA-exendin-4 PET/CT findings in patients with negative or inconclusive conventional imaging resulted in lesion-directed surgical planning and subsequent surgical intervention. In patients who underwent surgery based on [^68^Ga]Ga-DOTA-exendin-4 PET/CT findings, postoperative clinical outcome was assessed using the hypoglycemia score at follow-up as described above.

### Statistical analysis

All data were analyzed using IBM® SPSS® Statistics (version 28.0.1.0). Continuous variables are reported as median and range, and categorical variables as absolute numbers and percentages. Exact (Clopper–Pearson) 95% confidence intervals were calculated for proportions. Detection rate was defined as the proportion of [^68^Ga]Ga-DOTA-exendin-4 PET/CT-positive findings in patients with negative or inconclusive conventional imaging. Both histologically proven insulinoma and diffuse nesidioblastosis were considered true positive findings.

Baseline characteristics were compared between patients with negative or inconclusive conventional imaging, positive conventional imaging, and those without available conventional imaging. Continuous variables are reported as median (range) and were compared using the Kruskal–Wallis test. Categorical variables were compared using the χ^2^ test. For diagnostic accuracy analyses (sensitivity) histological assessment was regarded as the reference standard and analyses were restricted to patients with available histology. Wilcoxon signed-rank test was used for paired comparison of blood glucose levels prior to [^68^Ga]Ga-DOTA-exendin-4 PET/CT (baseline) and nadir during the examination (observation period: 2 hours). All statistical tests were two-sided, and a *P*-value < .05 was considered statistically significant.

## Results

### Patient characteristics and imaging

A total of 101 patients with biochemically proven EHH and/or positive Whipple's triad underwent [^68^Ga]Ga-DOTA-exendin-4 PET/CT at two tertiary referral centers. Blood glucose measurements during PET/CT were available in a subset of 52 patients and demonstrated a significant decrease during the examination (median baseline 5.6 vs nadir 3.6 mmol/L; *P* < .0001). Detailed baseline patient characteristics are summarized in Table 1.

**Table 1:** Patient characteristics

Study site	
University Hospital Freiburg	31
University Hospital Basel	70
Total	101
Age (years)	
Median (range)	57 (23-96)
Sex	
Male	55 (54.5%)
Female	46 (45.5%)
Body weight (kg)	
Median (range)	74 (46-133)
72-h fasting test available	81 (80%)
Blood glucose baseline prior [^68^Ga]Ga-DOTA-exendin-4 PET/CT (mmol/L)	
Median (range)	5.6 (3.0-13.6)
Blood glucose nadir after [^68^Ga]Ga-DOTA-exendin-4 administration (mmol/L)	
Median (range)	3.6 (1.1-9.0)

Blood glucose levels at baseline and at nadir during [^68^Ga]Ga-DOTA-exendin-4 PET/CT were available for 52 patients.

Prior conventional imaging comprised CT, MRI, SSTR PET/CT, [^18^F]FDOPA PET/CT, and EUS and was available in 87 patients. Among these, 58 patients showed negative or inconclusive findings and constituted the primary analysis cohort (Fig. 1). Details on the results of the performed conventional imaging modalities and their overlap are summarized in Table 2.

**Figure 1:**
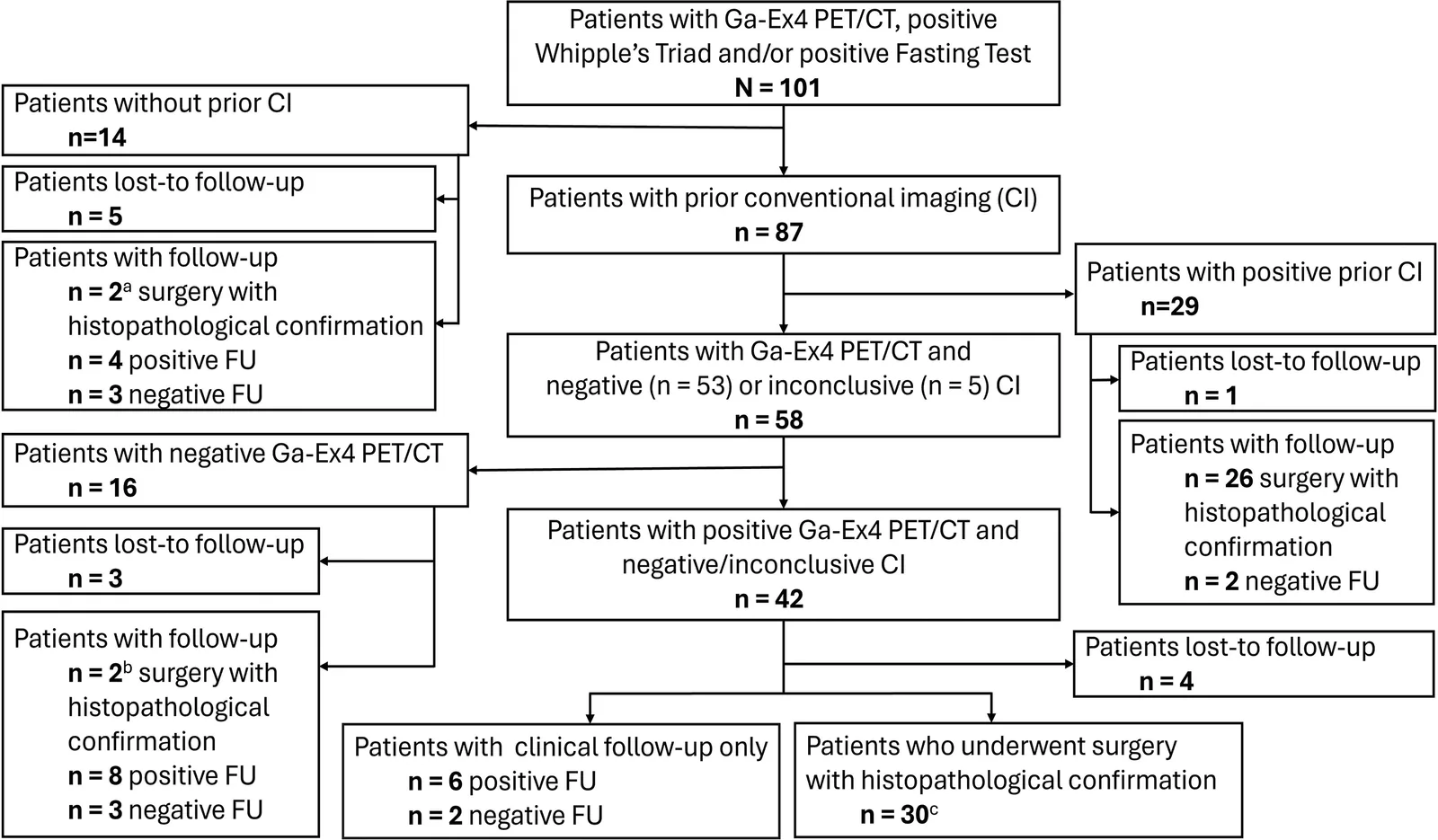
Study flowchart: Ga-Ex4 PET/CT = [^68^Ga]Ga-DOTA-exendin-4 PET/CT. Positive follow-up (FU) was defined as a hypoglycemia score of 1 or 2. Negative follow-up was defined as a hypoglycemia score of 0. ^a^Histology revealed benign insulinoma in both patients. ^b^Histology revealed diffuse nesidioblastosis in both patients. ^c^Histology revealed benign insulinoma (n = 29) and diffuse nesidioblastosis (n = 1).

**Table 2:** Conventional imaging findings

Imaging modality	Scans performed	Positive findings n (%)
CT	34	11 (32%)
MRI	60	16 (27%)
SSTR PET/CT	14	3 (21%)
FDOPA PET/CT	1	0 (0%)
EUS	22	6 (27%)
Positive conventional imaging was defined as detection of an insulinoma-suspicious lesion. Numbers reflect scans performed, not patients, as some patients underwent more than one modality.		
Overlap of positive conventional imaging findings		
Patients with prior conventional imaging		87
Patients with positive conventional imaging finding		29
Positive finding on one modality only		23
Positive finding on ≥2 modalities		6

Baseline characteristics stratified by prior conventional imaging status are presented in Table S1 (29). No statistically significant differences in baseline characteristics were observed between patients with negative or inconclusive conventional imaging, positive conventional imaging, and those without conventional imaging (all *P* > .05).

### Detection rate of [^68^Ga]Ga-DOTA-exendin-4 PET/CT

The study flowchart is illustrated in Fig. 1. In the primary analysis cohort, [^68^Ga]Ga-DOTA-exendin-4 PET/CT was positive in 42 patients and negative in 16 patients, corresponding to a detection rate of 72% (95% CI, 59-83%).

Representative imaging examples are shown in Figs. 2 and 3, illustrating (i) a focal GLP-1R-positive insulinoma not detectable on MRI or CT, and (ii) diffuse nesidioblastosis presenting with diffuse GLP-1R expression on PET/CT without a correlate on prior MRI.

**Figure 2:**
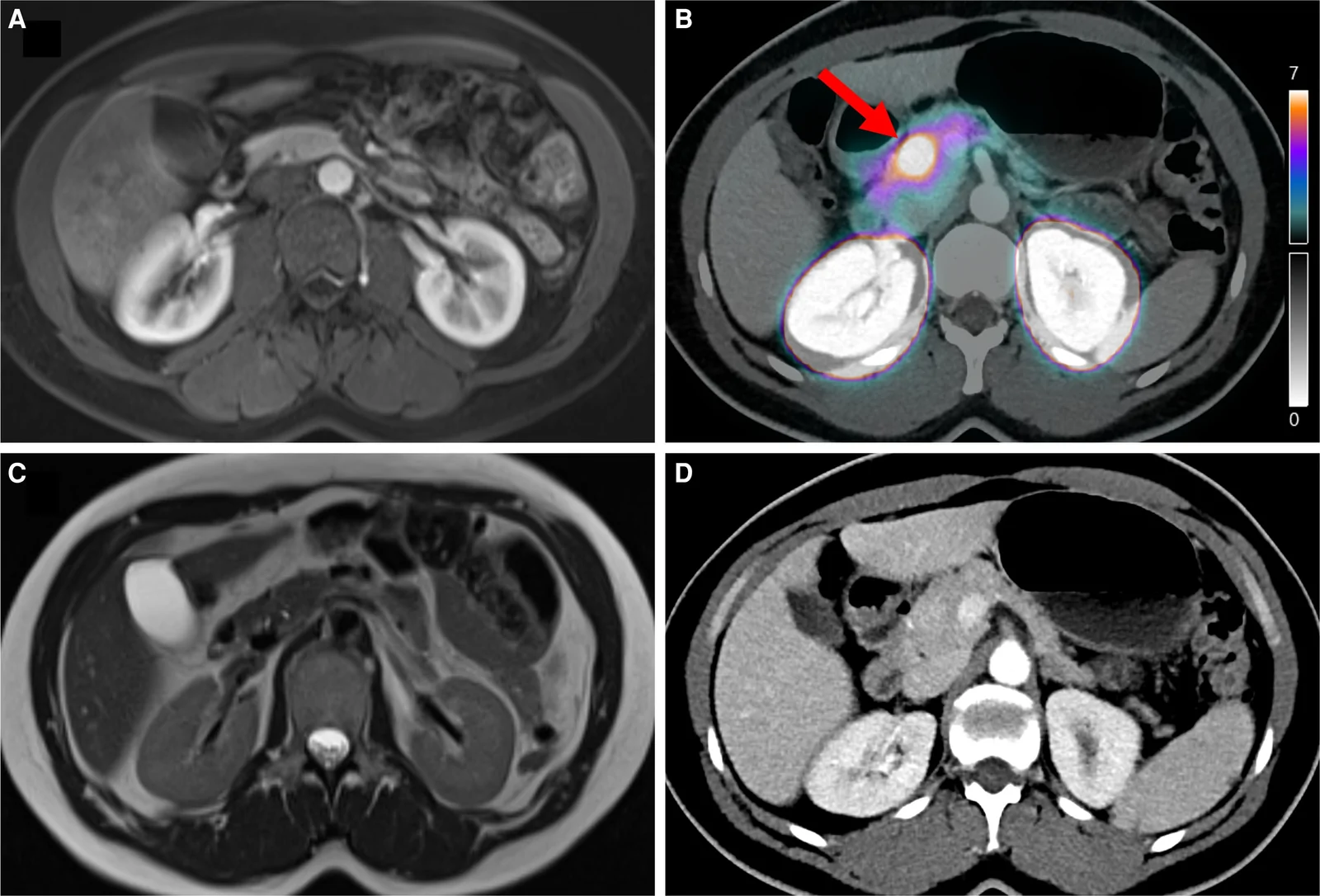
36-year-old female presenting with Whipple's triad and positive 72-h-fasting test. (A, C) MRI (T1 arterial phase and T2) shows no pancreatic lesion. (B) [^68^Ga]Ga-DOTA-exendin-4 PET/CT demonstrates intense focal tracer uptake corresponding to an insulinoma (red arrow), without a correlate on the contrast-enhanced CT (D). The patient underwent Whipple resection, and histopathology confirmed a benign, GLP-1-positive insulinoma (14 mm, G2, Ki-67 3%). PET/CT image (B) is displayed using a SUVmax color scale.

**Figure 3:**
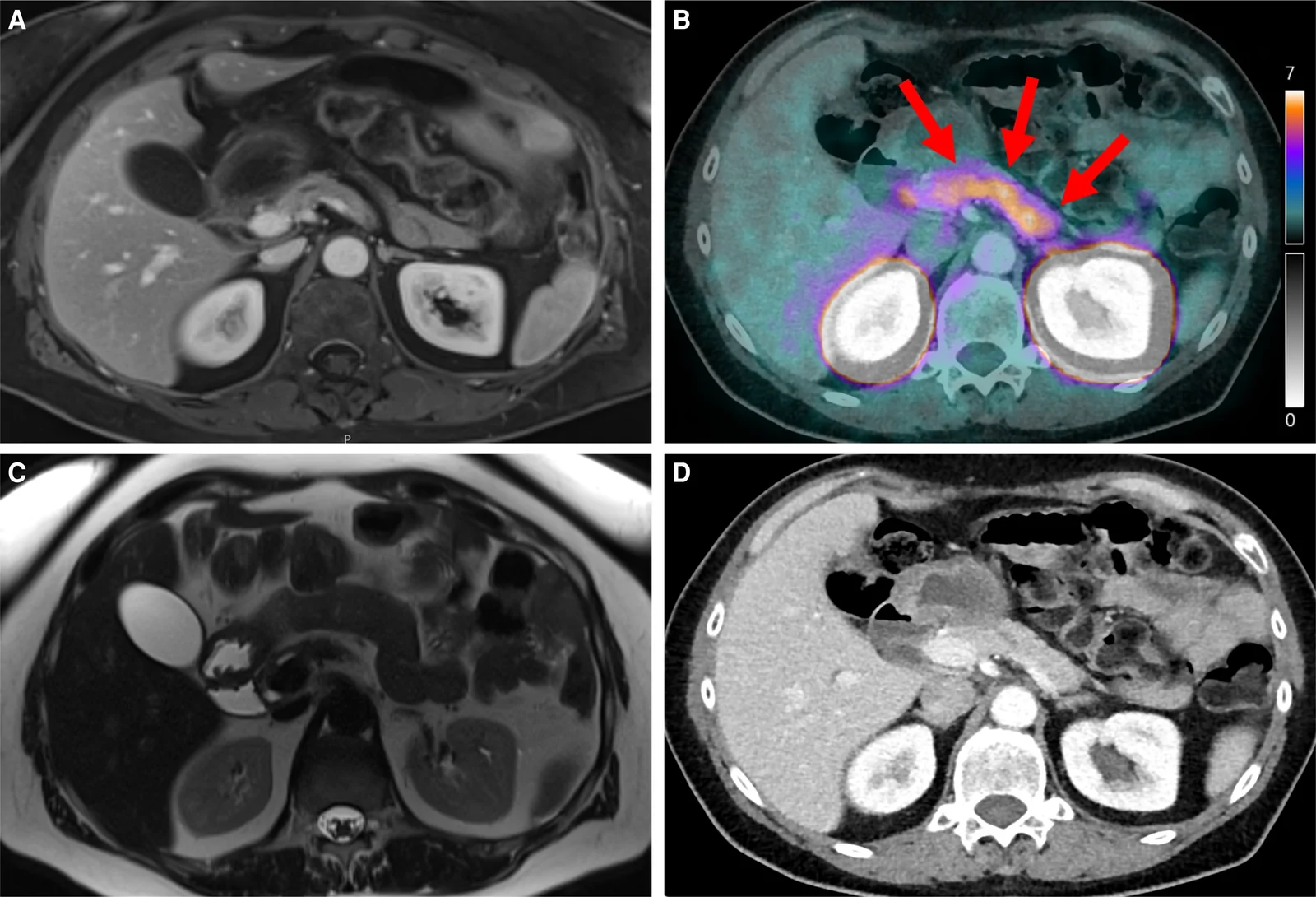
61-year-old female patient presenting with Whipple's triad and positive 72-h-fasting test. (A, C) MRI (T1 arterial phase and T2) shows no focal pancreatic lesion. (B) [^68^Ga]Ga-DOTA-exendin-4 PET/CT demonstrates diffuse GLP-1 receptor-mediated tracer uptake in the pancreas (red arrows), without a corresponding correlate on contrast-enhanced CT (D). The patient underwent total pancreatectomy, and histopathology confirmed diffuse nesidioblastosis. PET/CT image (B) is displayed using a SUVmax color scale.

### Clinical outcomes

Among the 42 patients with a positive [^68^Ga]Ga-DOTA-exendin-4 PET/CT finding, 4 were lost to follow-up. Of the remaining 38 patients, 30 underwent surgery with histopathological confirmation, revealing solitary insulinoma in 29 cases and diffuse nesidioblastosis in one case.

Eight patients were managed conservatively and assessed by clinical follow-up. Among them, the hypoglycemic score at follow-up was 0 (no hypoglycemic episodes) in two and 1 (conservatively manageable episodes) in six patients.

Among the 16 patients with a negative [^68^Ga]Ga-DOTA-exendin-4 PET/CT, three were lost to follow-up. Of the remaining 13 patients, two underwent surgery, both with histopathological confirmation of diffuse nesidioblastosis.

Eleven patients were managed conservatively and assessed by clinical follow-up. Among them, 3 experienced complete resolution of symptoms without further hypoglycemic episodes (hypoglycemia score = 0). All 3 had presented symptomatic hypoglycemia prior to PET/CT but had not undergone a fasting test. The remaining 8 patients showed persistent conservatively manageable episodes of hypoglycemia and were managed medically, most frequently with diazoxide (n = 3), alpelisib (n = 1), pasireotide (n = 1), diazoxide combined with low-dose corticosteroids (n = 1), or alimentation-controlled management (n = 1). One patient with known congenital hyperinsulinism received treatment with lanreotide and diazoxide.

### Diagnostic accuracy in histologically verified patients

Overall, 32 patients from the primary analysis cohort had histopathological verification (including two PET-negative cases). In this subgroup, [^68^Ga]Ga-DOTA-exendin-4 PET/CT demonstrated a sensitivity of 93.8% (95% CI, 79-99%), with no false-positive findings.

The two false-negative cases corresponded to diffuse nesidioblastosis, whereas all histologically confirmed insulinomas were correctly localized.

### Clinical impact on management

In 30 of 58 patients (52%) with negative or inconclusive conventional imaging, positive [^68^Ga]Ga-DOTA-exendin-4 PET/CT findings enabled lesion-directed surgical planning and led to surgical intervention. At follow-up, all of these patients were free of hypoglycemic episodes (hypoglycemia score = 0).

## Discussion

This international, dual-center, retrospective, real-world imaging study evaluated the diagnostic performance and clinical impact of [^68^Ga]Ga-DOTA-exendin-4 PET/CT in patients with EHH and negative or inconclusive conventional imaging, representing the most clinically relevant patient population for additional localization strategies.

In this primary analysis cohort (n = 58), [^68^Ga]Ga-DOTA-exendin-4 PET/CT demonstrated a detection rate of 72% and a sensitivity of 93.8% among histologically verified patients. Our detection rate is in line with recent real-world data by Aveline et al, who reported a detection rate of 63% using [^68^Ga]Ga-NODAGA-exendin-4 PET/CT in a comparable patient population (27). In terms of sensitivity, our findings are consistent with previously reported prospective data reporting sensitivities of approximately 94% for GLP-1R-targeted PET/CT for insulinoma localization (18, 25). When compared with other second-line localization techniques, invasive procedures such as ASVS have been reported to achieve sensitivities exceeding 85% (15-17). While ASVS provides indirect functional localization, GLP-1R-targeted PET/CT enables direct, noninvasive lesion visualization. In addition to ASVS, other second-line localization modalities such as EUS are commonly used in clinical practice. While EUS may achieve high detection rates in experienced centers, its performance is operator-dependent and may be limited in small or anatomically challenging lesions.

Beyond diagnostic performance, our findings highlight the substantial clinical impact of GLP-1R-targeted PET/CT in this difficult patient population. Positive [^68^Ga]Ga-DOTA-exendin-4 PET/CT findings enabled lesion-directed surgical planning in 52% of patients, with complete resolution of hypoglycemic symptoms in all operated cases. These results underscore the value of [^68^Ga]Ga-DOTA-exendin-4 PET/CT in facilitating curative, pancreas-preserving surgical approaches.

Importantly, all histologically confirmed insulinomas were correctly localized, underscoring the robustness of GLP-1R-targeted PET/CT in clinical practice. This is further substantiated by the low inter-reader variability reported for the GLP-1R-targeted PET/CT interpretation by board-certified nuclear medicine physicians as shown in previous studies (18). These findings further support the role of GLP-1R-targeted PET/CT as a second-line localization modality after inconclusive conventional imaging or EUS, consistent with ENETS guideline recommendations for GLP-1R-targeted imaging or ASVS in this clinical setting (26).

In our cohort, additional functional nuclear imaging modalities such as SSTR PET/CT or [^18^F]FDOPA PET/CT were performed in selected patients prior to GLP-1R-targeted PET/CT but provided limited additional diagnostic value in this cohort. In contrast, GLP-1R-targeted PET/CT provided definitive lesion localization in a substantial proportion of cases, highlighting its complementary role within the diagnostic pathway.

The two false-negative cases in our cohort corresponded to diffuse nesidioblastosis. In both instances, pancreatic tracer uptake did not exceed physiological levels, leading to a negative interpretation by the readers. In contrast, one patient with diffuse nesidioblastosis in our cohort displayed diffusely increased pancreatic uptake, allowing the diagnosis to be made. This illustrates that while GLP-1R-targeted PET/CT is highly accurate for insulinoma detection, its ability to diagnose nesidioblastosis remains limited and requires careful interpretation of subtle uptake patterns. Most likely, this is related to the heterogeneous biological background of this rare entity (30, 31).

Several limitations of our study must be acknowledged. First, some patients did not undergo a standardized fasting test but were included based on a positive Whipple's triad alone. While this reflects real-world clinical practice—where prolonged fasting may eg, not be feasible in frail patients—it may have introduced heterogeneity into the study population. Second, different PET/CT systems and acquisition protocols were used across the study sites, which may have introduced variability in image acquisition. However, the observation that sensitivity was comparable across the two institutions suggests that the diagnostic performance was largely driven by the GLP-1R-targeting ligand rather than by site-specific hardware or acquisition protocols. Third, while the retrospective design limited standardization of preimaging workup and follow-up, it also reflects real-world clinical practice and thereby enhances the generalizability of our findings. For example, MRI examinations performed in community radiology practices in both Switzerland and Germany may lack the advanced imaging protocols and technical standards of academic centers, and images may be interpreted by radiologists without dedicated subspecialty training. This is consistent with the fact that a standardized MRI protocol with dedicated board-certified readers in an academic institution considerably improves the sensitivity from 38% to 69% as previously shown (18). Fourth, there is a selection bias in the described cohort since conventional imaging tended to underperform compared with the published literature (13, 14, 32). This discrepancy might be explained by the fact that many of the patients in the study were referred following negative conventional imaging.

To our knowledge, this is the largest real-world cohort of patients undergoing [^68^Ga]Ga-DOTA-exendin-4 PET/CT for localization of insulinoma and nesidioblastosis in patients with EHH. Importantly, the study directly addresses patients with negative or inconclusive conventional imaging, who are most in need of improved localization strategies. In this group, [^68^Ga]Ga-DOTA-exendin-4 PET/CT localized predominantly insulinomas and one case of diffuse nesidioblastosis in 72% of patients, substantially reducing the need for invasive localization procedures and enabling pancreas-preserving surgical approaches or even local-ablative procedures. By enabling earlier and more accurate localization, GLP-1R-targeted PET/CT has the potential to shorten the time to curative surgery—a particularly important health-economic advantage in patients suffering from symptomatic hypoglycemia. Moreover, the ability to obviate invasive localization procedures such as selective arterial calcium stimulation with hepatic venous sampling (ASVS), which has been reported to achieve high sensitivities for insulinoma detection, can further reduce procedural risks while providing direct, noninvasive lesion localization.

Regarding safety considerations, GLP-1R-targeted PET/CT is usually well-tolerated with the occurrence of mainly gastrointestinal side effects, well-known in the context of GLP-1 analogs (33). Interestingly, hypoglycemia could be frequently observed after administration of [^68^Ga]Ga-DOTA-exendin-4, a finding that is usually not observed in the context of GLP-1 analog therapy in type 2 diabetes (34). The underlying mechanism for these findings remains to be elucidated but preliminary data indicate that exendin may be a valid alternative method to diagnose EHH replacing the cumbersome fasting test (35). However, in the context of GLP-1R-targeted PET/CT a parallel intravenous administration of glucose 10% is recommended or at least a regular monitoring of blood glucose levels during the procedure. This precaution is warranted because the injected amount of peptide may reach a pharmacologically active range—comparable to the microgram doses of GLP-1 analogs—and thus potentially aggravate hypoglycemia in patients with EHH. This association should be further elucidated in a future trial.

## Conclusion

This international, dual-center real-world study demonstrates the high diagnostic performance of [^68^Ga]Ga-DOTA-exendin-4 PET/CT for localizing insulinomas in patients with negative or inconclusive conventional imaging.

In this particularly challenging cohort, PET/CT achieved a detection rate of 72% and a sensitivity of 93.8% in histologically verified patients, with a substantial impact on clinical management by enabling pancreas-preserving surgical approaches.

By facilitating direct, noninvasive insulinoma localization, GLP-1R-targeted PET/CT has the potential to reduce the need for invasive diagnostic procedures and should be considered an important modality in the diagnostic pathway of patients with EHH.

## Data Availability

Original data generated and analyzed during this study are included in this published article or in the data repositories listed in References.
